# Secure near-lossless medical image compression and encryption with image-specific post-quantum key management and blockchain-based verification

**DOI:** 10.1371/journal.pone.0348013

**Published:** 2026-05-08

**Authors:** Bicky Yadav, Megha Arakeri

**Affiliations:** Manipal Institute of Technology Bengaluru, Manipal Academy of Higher Education, Manipal, India; University of the West of Scotland, UNITED KINGDOM OF GREAT BRITAIN AND NORTHERN IRELAND

## Abstract

Secure image transfer is a critical requirement in telemedicine and Picture Archiving and Communication Systems (PACS), where diagnostic integrity and patient confidentiality must be simultaneously ensured. High-resolution medical images, especially Magnetic Resonance Imaging (MRI), are often transferred over bandwidth-limited networks without impairing diagnostic quality or confidentiality against unauthorized access, manipulation, and replay. Existing approaches generally rely on a cascaded pipeline where image compression, encryption, and verification are considered as separate, independent operations. These distributed designs are prone to cryptographic key management issues, suffer from non-deterministic error control, lack meaningful coupling between image identity and cryptographic state, and provide no verifiable provenance or post-quantum key migration support. In this paper, we propose a DICOM-compliant system that integrates ϵ-regularized near-lossless image compression, hyperchaotic permutation diffusion encryption, post-quantum secure key establishment, and blockchain-based provenance verification as a unified operation. JPEG 2000 is used for primary image compression, and a residual refinement layer is introduced to limit residual correction amplitudes by ϵ-regularization to retain diagnostically significant information. Hyperchaotic encryption is performed with a symmetric image key generated using the NIST-standard post-quantum Key Encapsulation Mechanism (ML-KEM) and extracted using the HMAC-based Key Derivation Function (HKDF), cryptographically linked to DICOM image metadata. Message integrity is ensured using a keyed-hash message authentication code (HMAC), while a permissioned blockchain records only non-sensitive cryptographic metadata to enable verifiable provenance. Experimental results on clinical MRI and susceptibility-weighted imaging datasets demonstrate high reconstruction quality (PSNR: 46–51 dB, SSIM: 0.92–0.97), compression ratios ranging from 7.14:1 to 23.03:1 depending on modality and ϵ, and strong cryptographic properties (NPCR: 99.6%, UACI: 33.46%, entropy: 7.9993). The evaluation focuses on MRI-based modalities, reflecting the intended scope of this study.

## 1 Introduction

The Digital Imaging and Communications in Medicine (DICOM) standard specifies how images, along with corresponding image metadata, are formatted, archived, and transferred from within a Picture Archiving and Communication System (PACS). The DICOM standard is essential in ensuring that image pixels, patient details, imaging factors, and study keys are compatible with image scanners, reading workstations, and hospital computer systems [1]. Over the last three decades, DICOM has played a critical role in making imaging informatics, making it easy for healthcare institutions to interpret similar images from different devices. On the other hand, improved technologies for Magnetic Imaging Resonance (MRI) scans and Computerized Tomography (CT) scans have significantly improved spatial resolution, resulting in images that produce huge megabytes of data, which hospitals have to transmit without affecting clinical services. New risks also emerge with the manipulation of images, ransomware that affects imaging services, leakage of patient-institution links from DICOM images used for 3D reconstruction, computer-assisted surgery, development of models, artificial models, among others, used in images shared via DICOM standards, which continue to increase with images used for making 3D reconstructions, computer-assisted surgeries, models for developing artificial intelligence models [2,3].

It is often the case that current imaging workflows view tasks such as image compression, encryption, and verification as separate endeavours. While the aim of image compression is optimized for rate-distortion trade-off, the need to encrypt is often viewed as a subsequent step that is achieved independently of image structure or health care need. This often leads to real-world issues: too much compression could obscure subtle pathologies, while too much security could ensure that either latency is a problem or else it would be difficult to manage cryptographic keying, undermining health care workflow [4,5]. An important real-world lesson of imaging security breaches is that as long as vulnerabilities lie with key management, sharing, or generation, there is no inherent security issue with the encryption algorithms themselves. This is clearly made more difficult with respect to post-quantum cryptography (PQC), since widely deployed public-key cryptographic schemes such as RSA and elliptic-curve Diffie–Hellman (ECDH) are known to be vulnerable to efficient attacks by sufficiently capable quantum adversaries, motivating the need for post-quantum alternatives [6]. Transport layer security (TLS) together with traditional image compression techniques, such as JPEG 2000, has been commonly used to protect medical images during network transmission [5,7]. However, this approach only protects data during network transmission and does not maintain a cryptographic relationship between the image identity and encryption keys after the medical image is saved, shared, or reused in a PACS system [8]. Additionally, this approach uses session keys that are valid for a short period and not image-specific, thus limiting its application in long-term image integrity verification, individual image auditability, and post-quantum key management in distributed clinical settings [8].

To overcome the aforementioned limitations, this paper proposes a standards-compliant system that deals with image compression, encryption, authentication, and verification. This is achieved by an integrated system that is standards-compliant and addresses requirements related to maintaining image quality, overcoming key management difficulties, and performing image verification without revealing confidential information. The main contributions of this work are presented below:

- Epsilon-Regularized Residual Compression: A two-level compression scheme is used, where a base image is created through JPEG 2000 compression, and a refinement step for residual compression is applied for the remaining intensity errors. The residuals are limited to a fixed range (±ϵ) before compression, acting as a regularization step to reduce large magnitudes of correction and hence prevent large errors in the refinement step. While this method does not strictly enforce a per-pixel error bound, it follows the principle of bounded-error near-lossless compression as seen in JPEG-LS [9], allowing for consistent reconstruction quality while maintaining diagnostically useful image details.
- DICOM-bound, post-quantum–secure key establishment and lifecycle: In post-quantum cryptography, a key-encapsulation algorithm is used to produce a common value for a session. This common value is, in turn, used with a HKDF (key derivation function) to produce a fresh, new symmetric key for a different image with every iteration. This particular key is directly associated with the trio of DICOM keys: (StudyInstanceUID, SeriesInstanceUID, SOPInstanceUID), along with the SOP Class UID, such that it is definitively bound to the identity of a particular image. This is what ensures that a different key is appointed to every image, that the same key is never appointed to an image twice, and that no image’s key is usable for another image, especially for purposes of image decryption. This particular key is used to initialize the four-dimensional memristive hyperchaotic system, which is used for changes in position (permutation), as well as changes in values (diffusion) [6,10–12].It is emphasized that the post-quantum property applies to key establishment and image-specific key derivation, while the subsequent symmetric encryption and authentication primitives rely on established cryptographic constructions.
- Permutation and bit-plane-aware diffusion with in-band authentication: In this diffusion process, both the compressed streams, namely the image and the corresponding residual image, are encrypted by applying a rearrangement of the pixels based on a string derived from the key, followed by a manipulation of the pixels within their bit planes based on keys. The result is an encrypted signal which, with a small change in the keys, is entirely different and has a statistical uniformity. The HMAC is calculated on the encrypted signal along with the corresponding metadata, which is checked even before a single signal is decrypted, thereby not accepting modified/repeated signals [11,13].
- Non-secret ledger attestation: In order to facilitate verification without revealing private data, a non-secret ledger attestation system is used, which keeps only three things: a public-key fingerprint derived from a post-quantum–resilient key establishment process, a hash of the DICOM identification fields. There are no secret keys, patient information, or image pixels stored in this framework. As such, the non-secret ledger maintains a secure zone for image provenance and modification verification, while they are compatible with the conventional audit trails used in healthcare in a PACS system-compatible manner [14].

The above elements form a coherent framework that maintains quality, facilitates quantum-resistant keying, ensures in-band authenticity, and allows for verification with tamper-evidence. The proposed solution is expected to close the performance gap between image compression efficiency and the cryptographic strength demanded by modern PACS systems, especially in AI-assisted healthcare applications.

The remainder of this paper is organized as follows. Literature Review section presents a comprehensive review of related work. Proposed Methodology section outlines the design and implementation of our framework. Security Analysis section provides a detailed security analysis of the proposed framework. Experimental Setup and Results section presents the experimental setup and quantitative results. Discussion section discusses the findings, limitations and future work. Finally, Conclusion section concludes the paper.

## 2 Literature review

In hospitals, the imaging technique relies on the Digital Imaging and Communications in Medicine (DICOM) standard, which specifies image storage, transfer, and handling. The DICOM imaging technique uses globally consistent formats, transfer mechanisms, and numbering systems such as the “Study Unique Identifier,” “Series Unique Identifier,” and “Service-Object Pair (SOP) Instance Unique Identifier” that permit reliable communication from scanners, workstations, archives, and analysis systems for long-term storage, reporting, and secondary analysis [1]. Previous research has identified DICOM imaging workflows that progress from “archive management” to “three-dimensional reconstruction” to “artificial-intelligence preprocessing” [15,16]. The increased resolution in imaging devices, along with increased metadata, increases the need for structure and uniformity. Additionally, for image compression, wavelet-based JPEG 2000 has been used extensively because it is capable of multi-resolution decomposition with “region-of-interest” access that is necessary for radiology interpretation, which typically begins with the analysis of a small “region-of-interest” [5,7]. “Near-lossless” compression, such as JPEG-LS, has imposed a rigorous per-pixel error bound that is required for capturing subtleties in diagnostic imaging [9,17]. Imaging standards emphasize that “images needing compression must remain diagnostic quality” [4]. More contemporary machine learning/hybrid models promise improved efficiency [18,19] but lack codec-independent guarantees on the “maximum error bound,” which is paramount for safety acceptance within the healthcare industry. The auxiliary ϵ-regularized residual layer within our approach addresses these limitations by constraining residual magnitudes prior to entropy coding, resulting in controlled and stable reconstruction behaviour while remaining fully DICOM-compatible with existing JPEG 2000 systems that are already common in hospitals today, thus fitting within the healthcare sector’s emphasis on solutions that require “few changes to existing systems” within hospitals.

Another significant area of research is chaos-based image encryption. In the initial stages, research started with two-dimensional chaos-based mappings to conceal pixels [20,21], but later research formally characterized the need for robust anti-degradation requirements, such as large key space, high sensitivity to keys, high NPCR/UACI values, and resistance to statistical, differential, and other types of attacks. In addition, there are common vulnerabilities, which include repeated use of keys, resulting in a degradation of the encryption strength irrespective of the strength of the algorithm, which has been identified inadequately [13]. Most recent research has led to the development of hyperchaotic systems, memristive systems, which result in improved unpredictability and high-dimensional image adaptation [22,23], which, when surveyed, helps identify that there are inherent strengths and weaknesses in real-world implementations [24]. The problem with most is that there is a lack of inherent authentication, which means that the encrypted image can be modified without it being recognized, in which case most lack association with keys, leading to a situation where the encryption keys are reused unintentionally for different scans.

Secure establishment of keys is a significant activity in medical imaging systems, especially when they are used in networks and cloud infrastructure. Modern solutions isolate per-image working keys with distinct sessions; therefore, research recommends that post-quantum mechanisms such as ML-KEM [6] should be used to produce protected secrets that are impenetrable even to quantum computers. HKDF [10], which uses SHA-256 [12], provides a means of deriving multiple keys from a single secret session safely, while HMAC provides integrity verification, meaning that modification in ciphertext, meta-data, is recognized instantaneously [11]. Recommendations on NIST are critical in ensuring that keys are managed throughout the lifecycle of the image [25,26]. This is because, by associating keys with DICOM attributes, a distinct key is produced for the image, which means that they are not reused undetectably, thus following best practices in healthcare cyber-security. Permissioned ledgers such as Hyperledger Fabric increase robustness; hence, a means of keeping non-tamperable logs is provided, which is controlled [14]. Other solutions such as MedRec provide a means of transparency in patient-consent, patient-access workflows [27,28], but later research has increased application scope to imaging in healthcare systems, such as [29–31]. The use of patient or image data within a ledger might, however, pose confidentiality problems, hence the need for selective recording mechanisms. This is not an issue with our approach, which uses non-sensitive cryptographic summaries [32].

Recent studies have further extended chaos-based image encryption by incorporating advanced dynamical systems, learning-assisted chaotic generators, and hardware-realizable designs. ANN-assisted chaotic pseudo-random number generators and space-filling curve–based scrambling have been shown to improve diffusion and resistance to statistical attacks, while remaining computationally feasible for image encryption applications [33]. Hyperjerk and multistable chaotic systems, including those that can be implemented in circuits and FPGAs, have shown greater complexity, large key spaces, and strong sensitivity to initial conditions. These properties make them very desirable for secure image encryption [34–36]. In parallel, research has been conducted on integrity protection and privacy preservation for medical imaging using reversible watermarking and hash-based tamper detection, with a focus on ROI integrity during transmission and storage [37,38]. In addition, post-quantum cryptographic primitives, such as lattice-based NTRU cryptography, have been explored to protect medical images against quantum attacks [39]. However, existing solutions have been shown to only provide, at most, individual solutions for compression, encryption, integrity verification, or post-quantum security, without a unified framework that addresses all four requirements in a DICOM-aware manner for PACS systems.

Among these axes, there are some gaps. Usually, the development of compression and encryption is carried out separately. This results in inefficiencies and mismatches when combined in a medical setting. In most cases, image encryption methods disregard post-quantum security or the lifetime of keys generated from identifiers. Ledger-based solutions tend to store sensitive data in clear text, undermining the adoption by hospitals due to privacy regulations and operational risk. Prior work on compression has focused on shrinking the file size but not on secure end-to-end transmission; chaos-based methods enhance confusion and diffusion but at the expense of missing DICOM-aware key management and early authentication; ledger systems offer auditing but don’t attach verification to the cryptographic identity of every medical image. There’s also relatively little effort toward fusing all four aspects-compression, encryption, authentication, and verification-into a single workflow that harmonizes with current PACS infrastructure. We address the above gaps with a DICOM-aware framework that unifies compression, encryption, authentication, and verification within a single workflow compatible with existing PACS workflows. Motivated by these gaps, the following design objectives guide the proposed framework:

- ϵ-regularized fidelity control
- post-quantum and identifier-derived key lifecycle
- authentication-before-decryption with stronger diffusion and
- privacy-preserving ledger design.

Table 1 provides a qualitative comparison of representative prior works, focusing on design-level factors such as error determinism, key management, authentication capability, DICOM awareness, and privacy-preserving provenance.

**Table 1:** Summary of existing work.

Ref	Method & technique used	Key limitations identified	Relevance to this work / improvements
[5]	JPEG 2000 wavelet-based medical image compression with scalable bitstreams and optional ROI coding	No deterministic per-pixel error bound; no encryption; no identity awareness	Our ϵ-regularized residual refinement limits extreme correction amplitudes prior to entropy coding, while enabling seamless integration with encryption and identity-aware key management
[9]	JPEG-LS (LOCO-I) near-lossless compression using prediction and bounded residuals	Codec-specific; unsuitable for hybrid pipelines; lacks encryption	Our ϵ-regularized residual formulation provides codec-independent residual stabilization compatible with JPEG 2000, without relying on JPEG-LS–style deterministic error guarantees
[13]	Chaos-based cipher security analysis using NPCR, UACI, entropy, and key sensitivity	Key reuse vulnerabilities; weak diffusion; susceptibility to known attacks	Our framework meets the above-mentioned demands and prevents crucial key reuse through per-image, identifier-bound keys derived via standardized key encapsulation and derivation mechanisms.
[14]	Hyperledger Fabric for permissioned, tamper-evident audit logging	Not image-specific; no DICOM UID binding; no compression or encryption context	Only UID hashes and ciphertext digests are stored, enabling imaging-aware provenance without PHI exposure
[19]	Adaptive arithmetic coding with DWT quantization and RSA-based encryption	High computational cost; lacks authentication and identity-bound key management	Our key management replaces RSA with lightweight, image-specific symmetric keys derived via standardized key encapsulation and HKDF, preventing key reuse and enabling HMAC-based verification.
[20]	2D chaotic-map encryption using confusion and diffusion	Weak under known or chosen-plaintext attacks; no authentication or identity binding	Resolved via hyperchaotic encryption, DICOM-UID-bound keys, and HMAC authentication
[21]	Survey of chaos-based cryptography (logistic, cat, Lorenz maps, etc.)	Identifies instability, low-dimensional insecurity, and weak key generation	We employ a 4D hyperchaotic system seeded via HKDF for stability and secure key generation
[22]	4D memristive hyperchaotic map with histogram equalization for image encryption	No compression integration; no secure key management; no provenance	We integrate hyperchaos with compression, image-specific key lifecycle management, and ledger-based verification.
[23]	Multiple-image encryption using hyperchaos, SVD, and modified RC5	High computational complexity; unsuitable for real-time use; lacks provenance	Our solution is lightweight, codec-agnostic, and supports blockchain-based verification
[32]	Blockchain-based chaotic fractal encryption for medical images	No post-quantum security; no near-lossless compression; weak DICOM integration	Our framework integrates bounded-error compression, image-specific key lifecycle management, DICOM-aware identity binding, and ledger-based verification.

**Table notes.** Ref refers to the citation number in the reference list.

## 3 Proposed methodology

The suggested solution secures DICOM images throughout the entire life cycle, including image compression, encryption, transmission, and image reconstruction, while still being PACS-compatible. For every DICOM image, a normalization step followed by an ϵ-regularized near-lossless compression is applied. In this process, the image is divided into two components: a primary JPEG 2000 layer that represents the base image content [7], and a residual refinement layer in which correction amplitudes are limited within a predefined range (±ϵ) prior to encoding [9]. This residual regularization stabilizes refinement behaviour without enforcing a strict deterministic reconstruction error bound.

Finally, the system executes Protocol A: post-quantum–resilient key establishment and Per-Image Derivation. Based on the receiver’s post-quantum public key, the sender executes the key-encapsulation operation with respect to the module lattice (ML-KEM) to derive a shared secret without exchanging any symmetric key through the network [6]. ML-KEM is adopted in this work as a representative post-quantum key encapsulation mechanism due to its standardization by NIST and its intended role as a drop-in replacement for classical public-key key-establishment schemes such as RSA and ECDH. The proposed framework does not rely on ML-KEM–specific properties; instead, Protocol A is designed to remain agnostic to the underlying KEM. Any secure post-quantum KEM providing equivalent security guarantees can be substituted without altering the compression, encryption, or verification pipeline. A comparative evaluation of alternative post-quantum schemes is therefore outside the scope of this work. This shared secret is then acted upon by the HMAC-based Key Derivation Function (HKDF) [10], which is built on the SHA-256 hashing standard [12], to derive a session key seed. This session key seed is then expanded into a respective image key for each image, which is linked to the unique identifiers of the image (DICOM: Study UID, Series UID, or SOP Instance UID) such that each image gets a unique key. This is to ensure that both parties derive the same key independently without reusing any key.

The per-image key is used to seed a four-dimensional memristive hyperchaotic generator, which in turn seeds two processes on the two compressed streams, which rearrange byte values according to byte position and modify values on the bit planes. Hyperchaotic systems add a great amount of confusion, high sensitivity to the key, and close to random behaviour on the ciphertext, making the encrypted stream attack-resistant [13,22]. Since the encryption occurs after compression and is not dependent on the used codec, the system remains adaptable and thus is entirely compatible with JPEG 2000 as well as future image codecs. In order to ensure integrity, an authentication part is introduced within the encrypted block. The HMAC is calculated from a distinct subkey to the per-image key, which is appended to the ciphertext [11]. This is verified prior to the decryption process, ensuring that the receiver is able to confirm whether there has been manipulation of the message as soon as possible. In addition, a permissioned ledger is kept with merely three non-secret values: a hash of the sender’s post-quantum public-key fingerprint, a hash of the DICOM UID context, and a message digest of the ciphertext. This is carried out by making use of a system such as Hyperledger Fabric [14], so that since non-sensitive values are kept, patient data is not disclosed throughout the verification process. On the receiving part, the reverse is carried out. The receiver decapsulate a copy of the ML-KEM ciphertext, believing once again on retrieval from shared secret [6], calculates once again the per-image key making use of HKDF [10], verifies the HMAC part [11], verifies the ledger record [14], and, lastly, decodes the two streams of the compressed images. The primary JPEG 2000 image stream, together with the corresponding ϵ-regularized residual refinement, is reconstructed to achieve near-lossless image quality with bounded correction amplitudes, while maintaining diagnostically acceptable fidelity.

In summary, our system integrates ϵ-near-lossless compression [9], post-quantum–resilient key establishment [6], HKDF-based per-image key derivation [10,12], hyperchaotic encryption [13,22], and ledger-based verification [14] into a single easy-to-implement overall process. The figure, as well as the corresponding algorithms, that follow describe the sender and receiver procedures.

### 3.1 End-to-end workflow overview

**Fig 1:**
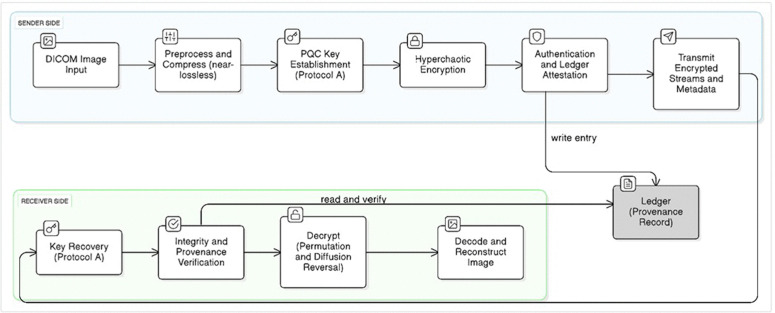
End-to-end step-by-step workflow of the proposed DICOM-compliant framework.

Fig 1 summarizes the complete end-to-end workflow of the proposed framework. At the sender side, each DICOM image is first normalized and compressed using an ϵ-regularized near-lossless scheme that produces two self-contained compressed outputs: a primary JPEG 2000 codestream and a primary JPEG 2000 codestream and a residual codestream produced under ϵ-regularized correction constraints applied prior to entropy coding. These compressed codestreams form the exclusive inputs to the cryptographic layer. Next, a post-quantum–resilient key establishment procedure (Protocol A) derives a shared session secret, from which an image-specific symmetric key is deterministically generated using DICOM identifiers. This per-image key initializes the hyperchaotic encryption engine, which independently encrypts the primary and residual codestreams using domain-separated sub-keys, without operating on raw pixel data. Before transmission, an authentication tag is computed over the encrypted streams and associated metadata, and a privacy-preserving verification record is written to a permissioned ledger. At the receiver side, the same cryptographic keys are re-derived locally, integrity and provenance are verified prior to decryption, and the encrypted codestreams are decrypted and decoded to reconstruct a near-lossless image consistent with the applied ϵ-regularized residual refinement.

Algorithms 1 and 2 present the complete sender- and receiver-side workflows implementing the proposed secure imaging framework. Protocol A defines the post-quantum–resilient session key establishment and the subsequent image-specific key derivation used by both sender and receiver.

**Notation Summary (used in Protocol A and Algorithms 1,2)**

- *pk*_*PQC*_, *sk*_*PQC*_: Receiver’s PQC public/private keys (e.g., ML-KEM).
- *ct*_*pqc*_: Ciphertext output from PQC encapsulation.
- shared_secret: KEM shared secret recovered after decapsulation.
- *S*: Session seed from HKDF (shared_secret, ...).
- *K*_*img*_: Per-image key derived from *S* and DICOM UIDs.
- $C_{primary}^{\prime },C_{res}^{\prime }$: Encrypted primary and residual streams.
- *Tag*: HMAC tag over canonical bundle.
- *LedgerTxnID*: Ledger transaction identifier for verification record.

**Protocol A — Secure Key Setup and Image-Specific Key Generation**

**Purpose:** Establish a post-quantum–resilient key establishment session, derive an image-specific symmetric key bound to DICOM identity, and deterministically seed the hyperchaotic generator, all without transmitting any symmetric keys.

**Inputs:** Sender: *pk*_*PQC*_, ReceiverID, nonce Receiver: *sk*_*PQC*_, *ct*_*pqc*_

**Outputs:** Shared session seed *S*, per-image key *K*_*img*_, chaos/keystream parameters

**Sender Side Steps:**

• A–S1: Encapsulate with *pk*_*PQC*_ to produce $\left(shared\_secret,ct_{pqc}\right)$

• A–S2: Derive *S* via HKDF(shared_secret, nonce, ReceiverID)

• A–S3: Derive *K*_*img*_ via HKDF(*S*, DICOM UIDs, SOP Class UID)

• A–S4: Map *K*_*img*_ to hyperchaotic generator parameters

**Receiver Side Steps:**

• A–R1: Decapsulate *ct*_*pqc*_ with *sk*_*PQC*_ to recover shared_secret

• A–R2: Re-derive *S* and *K*_*img*_ with identical HKDF labels

**Summary:** Both participants independently compute identical session and image-specific keys. No symmetric key ever leaves its origin, ensuring ensuring quantum-resilient key establishment confidentiality and deterministic linkage between cryptographic state and DICOM identity.

### 3.2 Sender-side framework

Algorithm 1 presents the sender-side workflow of the proposed secure DICOM imaging framework.

**Algorithm 1 Sender-side algorithm**

1:   **Goal:** Compress, encrypt, authenticate, and register verification for a DICOM image without transmitting any symmetric keys.

2:   **Inputs:** DICOM image *I*, PQC public key *pk*_*PQC*_, quality parameters *Q*_*p*_, *Q*_*r*_, error bound ε, receiver identifier, session nonce

3:   **Outputs:** Encrypted streams $C_{primary}^{\prime },C_{res}^{\prime }$, bit depth *B*, PQC ciphertext *ct*_*pqc*_, authentication tag *Tag*, ledger transaction ID *LedgerTxnID*

**Step 1 — Preprocess & Compress (**ε**-regularized near-lossless)**

4:   Load DICOM file and extract pixel data, bit depth *B*, and UIDs

5:   Normalize pixel intensities to [0,1]

6:   Encode primary JPEG 2000 stream with quality *Q*_*p*_

7:   Compute residual and clamp correction amplitudes to ±ε prior to entropy coding

8:   Encode residual stream using JPEG 2000 with quality *Q*_*r*_

**Step 2 — Key establishment (Protocol A)**

9:   Encapsulate with *pk*_*PQC*_ to obtain shared_secret and *ct*_*pqc*_

10:   Derive session seed *S* using HKDF with nonce and receiver identifier

11:   Derive per-image key *K*_*img*_ from *S* and DICOM UIDs

12:   Derive sub-keys via HKDF:

$K_{perm\_p},K_{diff\_p},K_{perm\_r},K_{diff\_r},K_{mac}$

13:   Initialize hyperchaotic generator seeded with *K*_*img*_

**Step 3 — Encrypt**

14:   Permute and diffuse primary stream using $K_{perm\_p}$ and $K_{diff\_p}$

15:   Produce encrypted primary stream $C_{primary}^{\prime }$

16:   Permute and diffuse residual stream using $K_{perm\_r}$ and $K_{diff\_r}$

17:   Produce encrypted residual stream $C_{res}^{\prime }$

**Step 4 — Authenticate & record**

18:   Define canonical *KDF*_*labels*_ including UIDs, parameters, and metadata

19:   Compute *Tag* using HMAC with key *K*_*mac*_

20:   Store ledger entry containing hashes of sender key, UIDs, and ciphertext

21:   Obtain ledger transaction ID *LedgerTxnID*

**Step 5 — Transmit**

22:   Send $\left\{C_{primary}^{\prime },C_{res}^{\prime },ct_{pqc},KDF_{labels},Tag,LedgerTxnID\right\}$ to receiver

23:   **Summary:** The sender performs ϵ-regularized near-lossless compression, post-quantum resilient key establishment, encryption, authentication, and verification recording with the constraints of diagnostic fidelity, confidentiality, and integrity verification.

#### 3.2.1 DICOM-aware pixel normalization.

Before ϵ-regularized near-lossless compression, the intensity values are normalized to the unit interval to provide a uniform interpretation of the error bound ϵ for all DICOM modalities and bit depths. Let *I*_*m*_(*x*, *y*) represent the original intensity values obtained from the DICOM file, and B represent the effective bit depth obtained from the BitsStored attribute. A linear, reversible normalization is applied:


$$I_{n}(x,y)=\frac{I_{m}(x,y)}{2^{B}-1},\;I_{n}(x,y)\in [0,1].$$ (1)

The normalization process does not include windowing, clipping, histogram equalization, and modality-specific rescaling. It preserves all relative intensity relationships and ensures that the ϵ parameter represents a physically meaningful bound on residual correction amplitudes prior to encoding, independent of image resolution or modality.

After decompression and reconstruction, the inverse operation is applied:


$$I_{\text{final}}(x,y)=round\;\left(I_{n}(x,y)\cdot (2^{B}-1)\right),$$ (2)

restoring the image to its original intensity scale. Because the normalization is strictly linear and fully reversible, it does not alter diagnostic contrast or clinically relevant intensity patterns. All quantitative fidelity metrics (PSNR and SSIM) are therefore computed in the normalized domain to enable fair comparison across images with different bit depths.

#### 3.2.2 Preprocessing and ϵ-Regularized Near-Lossless Compression.

Every image in the DICOM series $I(x,y)\in {\mathbb{C}}^{M\times N}$ is then broken down using predefined libraries for extracting pixel information and bit depth *B*, as well as other identifiers such as StudyUID, SeriesUID, SOPInstanceUID, and SOPClassUID. In the case of complex modalities like SWI, the real-valued magnitude image is extracted for simplifying image processing. Normalization of pixel values between 0 and 1 is done based on bit depth.

Compression is achieved through a two-step process:

- Primary JPEG 2000 compression yields a lossy, yet adequately diagnostic, base image.
- A residual map preserves the information lost in the base map, imposing a deterministic pixel-wise reconstruction constraint:
  $$R(x,y)=I_{n}(x,y)-I_{\text{decomp}}(x,y).$$ (3)
  $$\tilde{R}(x,y)=clip\;\left(R(x,y),-\epsilon ,+\epsilon \right).$$ (4)

Residual clipping is used to limit the possible correction amplitudes before entropy coding, acting as a regularization step to stabilize the reconstruction quality and prevent over-amplification of local errors. While it does not impose a hard deterministic error constraint after reconstruction, it provides a controlled refinement loop that has been observed to improve reconstruction accuracy for various image resolutions and modalities [5,7,9]. Specifically, the primary and residual codestreams are encoded separately into distinct JPEG 2000 bit streams for transmission.

#### 3.2.3 post-quantum–resilient key establishment (Protocol A).

A post-quantum Key Encapsulation Mechanism (KEM) such as ML-KEM [6] begins with the shared session secret initialization:


$$(ct_{\text{PQC}},\text{shared\_secret})=\text{Encapsulate}(pk_{\text{PQC}}).$$ (5)

From this, a session seed *S* is derived through HKDF [9]:


S=HKDF(shared_secret,salt=nonce,info=ReceiverID). (6)

followed by a per-image master key bound to DICOM identifiers:


$$K_{\text{img}}=\text{HKDF}(S,\text{info}=\{\text{StudyUID},\text{SeriesUID},\text{SOPInstanceUID}\}).$$ (7)

To avoid re-use of keystreams or correlation between domains, independent sub-keys are generated from *K*_img_ using domain-separated HKDF labels:


$$K_{\text{perm\_p}}=\text{HKDF}(K_{\text{img}},\text{info}=“\text{perm:primary}”),$$

$$K_{\text{diff\_p}}=\text{HKDF}(K_{\text{img}},\text{info}=“\text{diff:primary}”),$$

$$K_{\text{perm\_r}}=\text{HKDF}(K_{\text{img}},\text{info}=“\text{perm:residual}”),$$

$$K_{\text{diff\_r}}=\text{HKDF}(K_{\text{img}},\text{info}=“\text{diff:residual}”),$$

$$K_{\text{mac}}=\text{HKDF}(K_{\text{img}},\text{info}=“\text{mac}”).$$ (8)

These sub-keys drive both the primary and residual encryption streams, as well as the authentication phase, ensuring that key isolation is strict.

**ML-KEM parameter selection:** The proposed framework employs the ML-KEM-768 parameter set, as standardized by NIST, for post-quantum key establishment. ML-KEM-768 provides NIST security level 3, offering a balanced trade-off between quantum-resistant security, key size, and computational efficiency. The parameter selection is suitable for medical image transfer applications, which require strong long-term confidentiality without causing significant communication overhead. In particular, ML-KEM-768 offers quantum resistance with the ability to maintain good performance for per-image key establishment in the PACS and tele-radiology environments. The post-quantum service is only used for symmetric key establishment, while the protection of bulk image data relies on efficient symmetric encryption.

#### 3.2.4 Hyperchaotic encryption.

This encryption engine uses a four-dimensional memristive hyperchaotic system [22–24] which is initiated with *K*_img_:


$$\dot{x}=a(y-x)+w+exz,$$

$$\dot{y}=bx-xz+cy,$$

$$\dot{z}=xy-dz,$$

$$\dot{w}=-x-ew.$$ (9)

whose discretized evolution yields the pseudorandom keystream:


$$k_{i}=\left(\left|10^{5}x_{i}+10^{4}y_{i}+10^{3}z_{i}+10^{2}w_{i}\right|\mathrm{mod}256\right),\;i=1,\ldots ,MN.$$ (10)

The primary code stream uses $\left(K_{\text{perm\_p}},K_{\text{diff\_p}}\right)$, while the residual uses $\left(K_{\text{perm\_r}},K_{\text{diff\_r}}\right)$. Each stream is then subjected to chaotic permutation followed by bidirectional diffusion, thereby ensuring high entropies, pixel-level key sensitivities, and lack of statistical correlations between neighboring pixels.

#### 3.2.5 Codec independence and syntax preservation.

The proposed encryption layer is designed to be codec-agnostic and does not change the syntax of the codec or the bitstream format. The compression is fully done before encryption, and this results in standards-compliant JPEG 2000 codestreams for both the primary and residual streams. The encryption process operates on the fully compressed codestream byte sequences and is fully reversible, such that all codec headers, markers, packet structures, and entropy coding constraints are restored exactly after decryption. At the receiving end, the decryption process is done before any decoding process, and this ensures that the JPEG 2000 decoder receives a valid and unaltered codestream. Therefore, the proposed encryption layer does not interfere with codec parsing after decryption and does not introduce any syntax violations in the decoded bitstream. The proposed framework is codec-independent by design and can be applied to other image codecs, provided that encryption is performed after compression and decryption precedes decoding.

#### 3.2.6 Authentication and ledger attestation.

To provide tamper-evident integrity and verification, both encrypted codestreams are authenticated with the special key *K*_mac_. All non-secret context parameters, such as session IDs, compressed parameters, and UIDs, are packaged into a standardized bundle:


$$\text{KDF\_labels}=\{\text{alg\_version},\text{ReceiverID},\text{nonce},\text{StudyUID},\text{SeriesUID},\text{SOPInstanceUID},\text{SOPClassUID},B,\epsilon ,Q_{p},Q_{r}\}.$$   (11)

Sender calculates the authentication tag:


$$\text{Tag}=\text{HMAC}\left(K_{\text{mac}},\{C_{\text{primary}}^{\prime },C_{\text{res}}^{\prime },\text{KDF\_labels}\}\right),$$ (12)

which ties the ciphertexts to any related parameters in such a manner that it is impossible to make any changes undetected in the compression quality, error tolerance, or study identifier. A permissioned ledger contains merely the non-secret information: public key fingerprint for PQC, context hash of the UID, or the ciphertext digest, establishing a privacy-preserving verification path that is compatible with existing PACS systems [14].

#### 3.2.7 Transmission.

The transmitted bundle,


$$\{C_{\text{primary}}^{\prime },C_{\text{res}}^{\prime },\text{KDF\_labels},ct_{\text{PQC}},\text{Tag},\text{LedgerTxnID}\},$$ (13)

is self-contained and verifiable. The receiver can independently re-derive the same cryptographic context and validate both the tag and the ledger entry before decryption. This sender-side workflow thus unifies ϵ-regularized near-lossless compression, post-quantum keying, hyperchaotic encryption, and verifiable verification into a single, standards-aligned imaging framework.

### 3.3 Receiver-side framework

**Algorithm 2 Receiver-side algorithm**

1: **Goal:** Verify authenticity, reconstruct keys locally, decrypt, and recover a high-fidelity reconstructed image.

2: **Inputs:** $C_{\text{primary}}^{\prime }$, $C_{\text{res}}^{\prime }$, *ct*_*pqc*_, *KDF*_*labels*_, *Tag*, *LedgerTxnID*, receiver’s private key *sk*_*PQC*_

3: **Output:** Reconstructed image *I*_final_

**Step 1 — Key Recovery (Protocol A)**

4: Decapsulate *ct*_*pqc*_ using *sk*_*PQC*_ to recover shared_secret

5: Recreate session seed *S* from shared_secret using HKDF with nonce and ReceiverID

6: Recreate per-image key *K*_img_ from *S* using HKDF with Study UID, Series UID, SOP Instance UID, and SOP Class UID

7: Derive domain-separated sub-keys:

$K_{\text{perm\_p}},K_{\text{diff\_p}},K_{\text{perm\_r}},K_{\text{diff\_r}},K_{\text{mac}}$

8: Recreate chaos parameters from *K*_img_

**Step 2 — Integrity & Verification**

9: Generate $Tag^{\prime }$ using HMAC with key *K*_mac_ over encrypted streams and labels

10: **if** $Tag^{\prime }\neq Tag$ **then**

11:   **Abort**

12: **end if**

13: Retrieve ledger record using *LedgerTxnID* and verify context hash and ciphertext digest

14: **if** ledger mismatch **then**

15:   **Abort**

16: **end if**

**Step 3 — Decrypt**

17: Reverse bit-plane diffusion on primary stream using $K_{\text{diff\_p}}$

18: Reverse permutation on primary stream using $K_{\text{perm\_p}}$

19: Reverse bit-plane diffusion on residual stream using $K_{\text{diff\_r}}$

20: Reverse permutation on residual stream using $K_{\text{perm\_r}}$

21: Obtain original JPEG 2000 codestreams

**Step 4 — Decode & reconstruct**

22: Decode primary and residual streams

23: Reconstruct normalized image *I*_norm_ = base + residual

24: Denormalize to bit depth *B* and clamp to valid range

**Step 5 — Return**

25: Output *I*_final_ as the verified, near-lossless image

The receiver module is responsible for the secure reconstruction of the DICOM image. This is governed by the strict “verify before decrypt” policy, where no computational work is involved in decrypting until it is established that the received bundle is authentic, untampered with, and received from the right source. This deterministic re-derivation of the cryptographic context validates integrity, decrypts the streams, and reassembles the reconstructed image refined by an ϵ-regularized residual.

#### 3.3.1 Post-quantum key decapsulation (Protocol A).

When the message bundle is received, the first operation of the receiver is to derive independently the whole cryptographic context. Using its private key *sk*_*PQC*_ and the received ciphertext *ct*_*PQC*_, the receiver decapsulates the KEM [6] to recover the identical shared session secret:


$$\text{shared\_secret}=\text{Decapsulate}(sk_{PQC},ct_{PQC}).$$ (14)

Using the shared_secret and the session parameters from the received *KDF*_*labels*_ bundle (i.e., nonce, ReceiverID), the receiver executes the identical HKDF [10] step from Eq. (6) to re-derive the session seed *S*. Next, using *S* and the DICOM identifiers from *KDF*_*labels*_, the receiver re-derives the per-image master key *K*_img_ identically to Eq. (7). Finally, it re-derives the complete set of domain-separated sub-keys by executing the same HKDF expansion from Eq. (8):


$$K_{\text{perm\_p}}=\text{HKDF}(K_{\text{img}},\text{info}=“\text{perm:primary}”),$$

$$K_{\text{diff\_p}}=\text{HKDF}(K_{\text{img}},\text{info}=“\text{diff:primary}”),$$

$$K_{\text{perm\_r}}=\text{HKDF}(K_{\text{img}},\text{info}=“\text{perm:residual}”),$$

$$K_{\text{diff\_r}}=\text{HKDF}(K_{\text{img}},\text{info}=“\text{diff:residual}”),$$

$$K_{\text{mac}}=\text{HKDF}(K_{\text{img}},\text{info}=“\text{mac}”).$$ (15)

At this stage, the receiver possesses the same set of six symmetric keys as the sender, without any of them having been transmitted.

#### 3.3.2 Integrity and verification.

Before any decryption is attempted, the receiver performs two mandatory verification checks. First, it validates the in-band authentication tag. The receiver locally computes its own HMAC [11] over the received data using the derived key *K*_mac_:


$$Tag^{\prime }=\text{HMAC}(K_{\text{mac}},\{C_{\text{primary}}^{\prime },C_{\text{res}}^{\prime },KDF_{labels}\}).$$ (16)

The calculated $Tag^{\prime }$ is then compared with the received Tag. If $Tag^{\prime }\neq Tag$, it implies that the message has been tampered with or corrupted, and the process is immediately aborted. Next, the receiver performs provenance verification based on the *LedgerTxnID*. The receiver queries the permissioned ledger [14], extracts the stored record, and verifies its authenticity. The receiver locally recalculates the UID-context hash and ciphertext digest, which are then compared with the stored record on the ledger. Any mismatch implies that the image is not the expected image or its origin is not trusted, and the process is aborted.

#### 3.3.3 Hyperchaotic decryption.

Only after the successful execution of the integrity and verification checks is the system moved to the decryption phase. The receiver reinitializes the same four-dimensional memristive hyperchaotic map [22–24] with the same key *K*_img_ to produce the same pseudorandom keystreams. The decryption process is the exact reverse of Section 3.2.4. It is performed independently for each stream. For the primary stream, the inverse bidirectional diffusion is performed using the keystream from $K_{\text{diff\_p}}$, followed by the inverse permutation using the keystream from $K_{\text{perm\_p}}$. For the residual stream, the inverse operations are performed using the keystreams from $K_{\text{diff\_r}}$ and $K_{\text{perm\_r}}$. This process deterministically produces valid and unaltered JPEG 2000 primary and residual codestreams ready for standard decoding.

#### 3.3.4 Image reconstruction.

Finally, the receiver decodes both codestreams using a standard JPEG 2000 decoder [5,7]. The final normalized image, *I*_recon_(*x*,*y*), is reconstructed by summing the base and residual layers:


$$I_{\text{recon}}(x,y)=I_{\text{decomp}}(x,y)+R_{\text{res}}(x,y).$$ (17)

This image is then denormalized using the bit depth *B* (from the *KDF*_*labels*_ bundle) to produce the final image, *I*_final_. Because residual values are constrained prior to encoding, the reconstruction error is strongly regularized and empirically bounded, resulting in stable near-lossless behavior with high fidelity, though without enforcing a strict deterministic per-pixel error bound after decoding.

### 3.4 Experimental reproducibility notes

To ensure that it is conducive to the reproduction of the study, the implementation details used in this study are explicitly listed in Section 5. A Python-based implementation of the entire procedure, from ϵ-regularized near-lossless image compression to post-quantum–resilient key establishment, hyperchaotic encryption, and verification processes, was accomplished using standard Python libraries for parsing the image data, JPEG 2000 image encoding, numerical computation, and metric analysis. All experiments were carried out using publicly accessible MRI image data with fixed resolution and bit depth, and with fixed compression and cryptographic parameters within each experimental configuration, hyperchaotic map initialization, PQC encapsulation, or authentication. These factors, including data description, hardware setup, metric definition, and fixed parameters for reproduction of the study, are provided in explicit detail in Section 5 without any need to refer to any black-box tool or intellectual property involved.

## 4 Security analysis

This section will discuss the security characteristics of the proposed framework from a system-level viewpoint that is relevant to medical imaging. The analysis focuses on confidentiality, integrity, and resilience against frequent cryptanalytic attacks. The research is empirical and does not attempt to provide formal cryptographic proofs but instead evaluates the framework’s behavior based on clearly defined and pragmatically justified assumptions about threats.

### 4.1 Threat model and security assumptions

The proposed framework is evaluated in a system-level threat model that matches real-world scenarios of PACS-based medical imaging. The protected resources include the pixel information of medical images, DICOM identifiers, and cryptographic keys used for encryption and authentication. The attacker is assumed to have access to the encrypted medical images, network communication channels, and the stored ciphertexts. The adversary does not have access to secret cryptographic keys or the internal state of the encryption process. The security objectives of this work are to preserve image confidentiality, enable integrity verification prior to decryption, and empirically evaluate robustness against ciphertext-only, known-plaintext, and limited chosen-plaintext attack scenarios. Formal cryptographic proofs, side-channel attacks, denial-of-service attacks, and ledger-level adversarial behaviors such as rollback or fork attacks are considered outside the scope of this study.The security analysis assumes a secure post-quantum key encapsulation mechanism for establishing shared secrets between communicating parties. ML-KEM is used in this work as a representative NIST-standardized post-quantum KEM; however, the proposed framework does not rely on any ML-KEM–specific structural properties. The confidentiality, integrity, and provenance guarantees analyzed in this section depend only on the existence of a quantum-resistant shared secret, and remain unchanged if ML-KEM is replaced with another secure post-quantum KEM. A comparative performance or security evaluation of alternative post-quantum schemes is therefore considered outside the scope of this study. The threat model explicitly includes replay of previously observed ciphertexts and potential misuse or collision of DICOM UIDs, which are mitigated through per-image key derivation, integrity verification, and ledger-based provenance mechanisms described below.

### 4.2 Ciphertext-only statistical analysis

**Fig 2:**
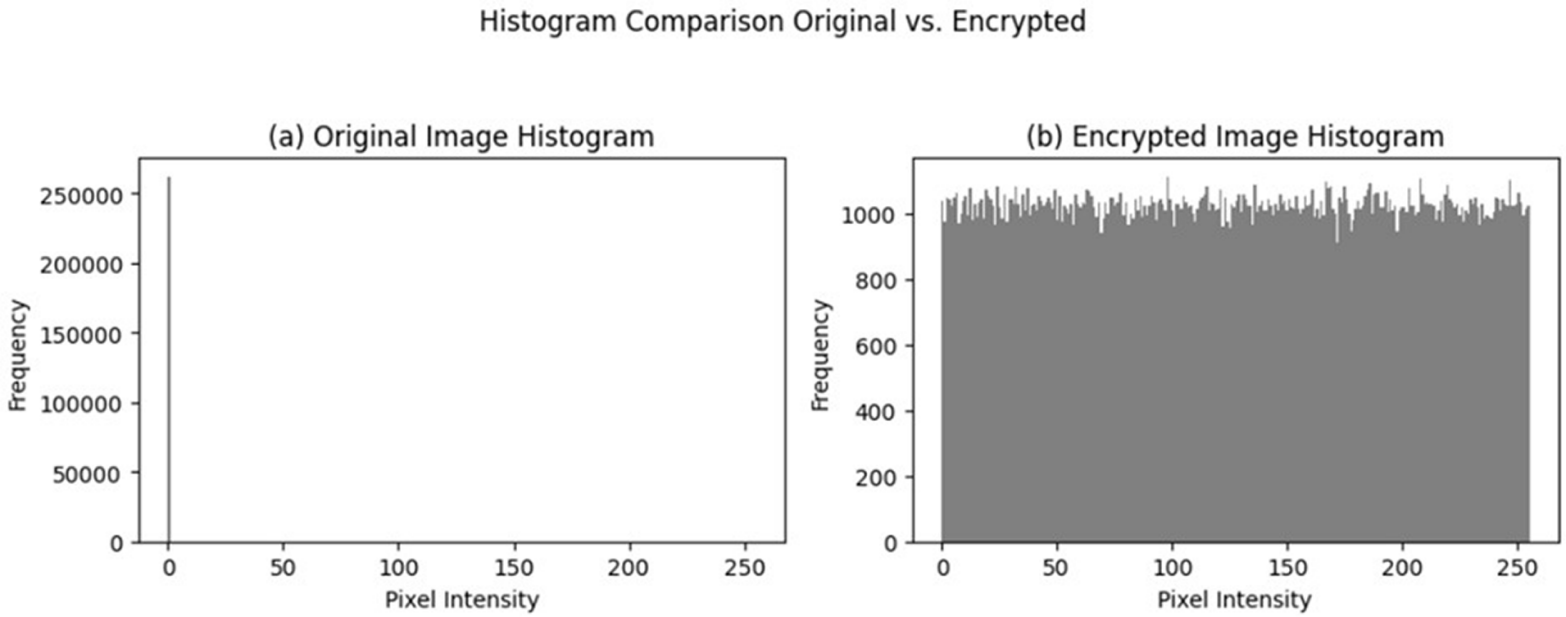
Histogram comparison between (a) the original medical image and (b) the corresponding encrypted image.

In the ciphertext-only attack scenario, the attacker aims to obtain information about the original image from the ciphertext without access to the plaintext. To test the robustness of the encrypted images against ciphertext-only attacks, the encrypted images were analyzed using standard statistical measures such as intensity histogram and entropy. The histogram of the encrypted images show in Fig 2 follows a uniform distribution, which is unstructured compared to the histogram of the original images. The unstructured histogram of the encrypted image implies that first-order intensity statistics and structural patterns are well hidden by the encryption process. Additionally, the entropy of the encrypted images is close to the maximum possible value, which implies high uncertainty and resistance to simple statistical analysis. These results imply that statistical analysis of ciphertext alone does not provide any useful information about the original medical image content.

The encrypted image has a near-uniform distribution, which is a clear indication of the successful suppression of the plaintext statistical features.

### 4.3 Randomness analysis using NIST SP 800-22

To further evaluate the statistical properties of the encrypted image data, a set of tests from the NIST SP 800−22 randomness test suite was applied to the ciphertext bitstreams obtained from the encrypted medical images (Table 2). Although the NIST SP 800−22 test suite was designed to test pseudorandom number generators, rather than image-derived ciphertexts, it has been widely used in the literature on image encryption as an empirical statistical measure of randomness. The following tests were applied to the bitstreams at a significance level of α=0.01: Frequency (Monobit) Test, Block Frequency Test, Runs Test, Approximate Entropy Test, and Serial Test. The bitstreams passed all the test criteria, such as the Monobit, Runs, Approximate Entropy, and Serial tests, which indicate that the bitstreams are empirically balanced and lack significant short-range statistical dependencies in the tested ciphertext bitstreams. The Block Frequency test showed a marginal failure, as reported in the image encryption literature, where the Block Frequency test is highly sensitive to the statistical variations in the plaintext image data. The marginal failure does not contradict the diffusion process, as the Block Frequency test is known to be sensitive to the structural correlations in image-derived bitstreams. The results indicate that the proposed encryption scheme produces ciphertext exhibiting strong statistical randomness characteristics under multiple NIST SP 800−22 tests, which is consistent with the differential security results (NPCR, UACI, and entropy) and further confirms that the encryption process successfully suppresses the plaintext statistical features.

**Table 2:** NIST SP 800−22 randomness test results for encrypted image bitstreams (α=0.01).

Test	p-value	Result
Frequency (Monobit)	0.0326	Pass
Block Frequency	<10^−3^	Fail
Runs	0.1878	Pass
Approximate Entropy	0.0278	Pass
Serial	0.0427	Pass

### 4.4 Scope of comparison with standard DICOM security mechanisms

Standard medical image security mechanisms specified in DICOM PS3.15 are largely based on traditional block cipher algorithms such as AES, typically used in authenticated encryption schemes such as AES-GCM [40]. These security mechanisms encrypt the DICOM bitstream as a whole and do not work with spatial image data or pixel-level image structure. Therefore, image-differential security metrics such as Number of Pixel Change Rate (NPCR) and Unified Average Changing Intensity (UACI) cannot be used in AES-based DICOM security profiles, as these metrics are designed to assess diffusion and confusion capabilities of image-aware encryption schemes. The proposed framework is not meant to replace standard AES-based DICOM security, but rather to complement it by adding image-aware encryption properties, post-quantum secure key establishment, and verifiable provenance. In this context, the proposed image-aware encryption scheme can coexist with existing AES-based DICOM transport security without requiring any changes to the existing PACS or DICOM infrastructure.

### 4.5 Known-plaintext attack analysis

**Fig 3:**
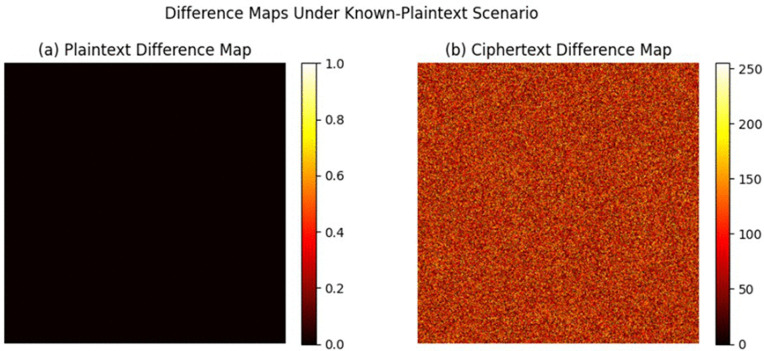
Difference maps for plaintext and ciphertext under a known-plaintext attack. **(a)** Difference map for two plaintext images differing by a single-pixel change, showing negligible differences. **(b)** Difference map for the corresponding ciphertext images, showing extensive and random variations throughout the entire image.

The known-plaintext attack scenario was considered, where the attacker has available one or more pairs of plaintext and ciphertext. In the context of medical imaging systems, this could occur if similar images or previous images of the same patient are available. To test the robustness in this scenario, two different plaintext images with a minimal pixel-level difference were encrypted using the same system. The corresponding ciphertexts were then compared using the Number of Pixel Change Rate (NPCR) and Unified Average Changing Intensity (UACI) measures. High NPCR and UACI values were found (NPCR ≈ 99.6%, UACI ≈ 33.4%), which indicates high sensitivity of the ciphertext to small changes in the plaintext. This phenomenon is also shown in Fig 3, where a negligible difference in the plaintext causes a large number of uncorrelated changes in the encrypted image. These findings confirm the diffusion and confusion properties of the proposed encryption system, which makes it very difficult for an attacker to deduce any information about the encryption process or the keys used, within the evaluated known-plaintext attack setting.

### 4.6 Limited chosen-plaintext attack analysis

In a limited chosen-plaintext attack, the attacker is assumed to have the capability to choose input images of their choice and analyze the resulting ciphertext images. In the context of medical imaging systems, such attacks may be launched through compromised input interfaces or repeated attacks on the system with carefully designed inputs. To assess the system’s resilience against this attack, structured plaintext images, including uniform images and images with locally controlled perturbations, were encrypted using the proposed system. Unlike in the known-plaintext attack, the attacker has the capability to choose the input images with the aim of exploiting structural or differential weaknesses. The resulting ciphertext images showed high sensitivity to the chosen plaintext images, with NPCR and UACI measures similar to those in the known-plaintext attack analysis.This indicates that, under the evaluated conditions, the ciphertext exhibits strong sensitivity to chosen plaintext variations, limiting the practical exploitability of structural or differential information. This analysis indicates that the proposed encryption system has practical resilience against limited chosen-plaintext attacks in the context of medical imaging systems.

### 4.7 DICOM UID collision–based key sensitivity analysis

**Fig 4:**
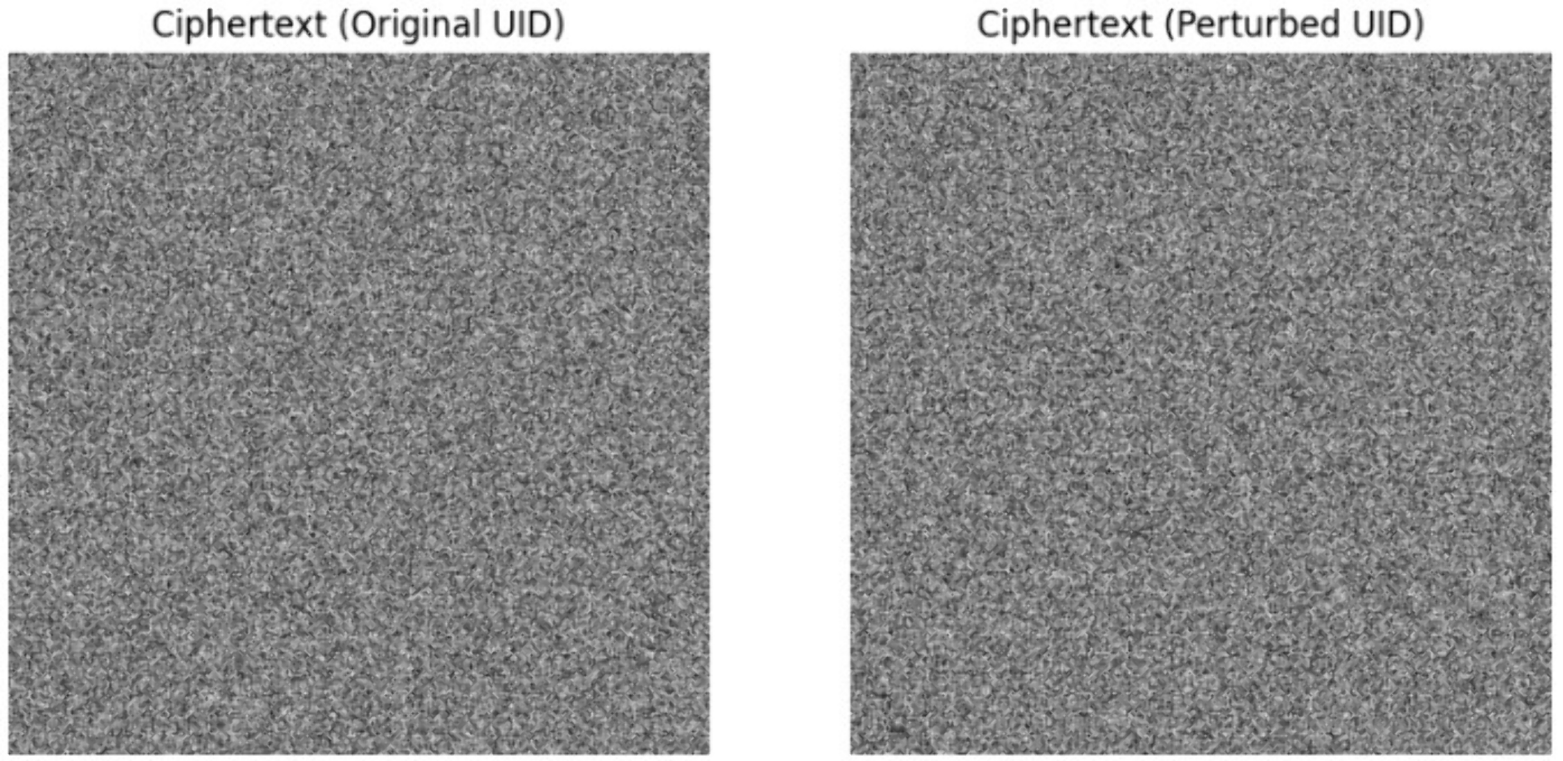
DICOM UID collision-based key sensitivity analysis. The ciphertexts produced from the same compressed medical image with the same system parameters show substantial and statistically uncorrelated differences, only differing by a single digit in the SOPInstanceUID.

To evaluate the sensitivity of the proposed per-image key derivation scheme to DICOM identifiers, a UID collision-based sensitivity attack was performed. Two encryption processes were carried out on the same compressed image with all parameters remaining unchanged, except for a single-digit variation in the SOP Instance UID. The generated ciphertexts shown in Fig 4 demonstrated close-to-ideal differential properties, with NPCR = 99.60% and UACI = 33.42%, indicating that even the slightest variation in DICOM UIDs leads to cryptographically independent encryption results. This verifies that the per-image key derivation scheme is heavily dependent on DICOM identifiers and is robust against accidental or intentional DICOM UID collisions. Since the replayed ciphertexts are still linked to the original DICOM UID-derived key information, any successful replay or substitution attack are effectively mitigated under the assumed threat model, including ciphertext replay scenarios.

### 4.8 Residual stream confidentiality

**Fig 5:**
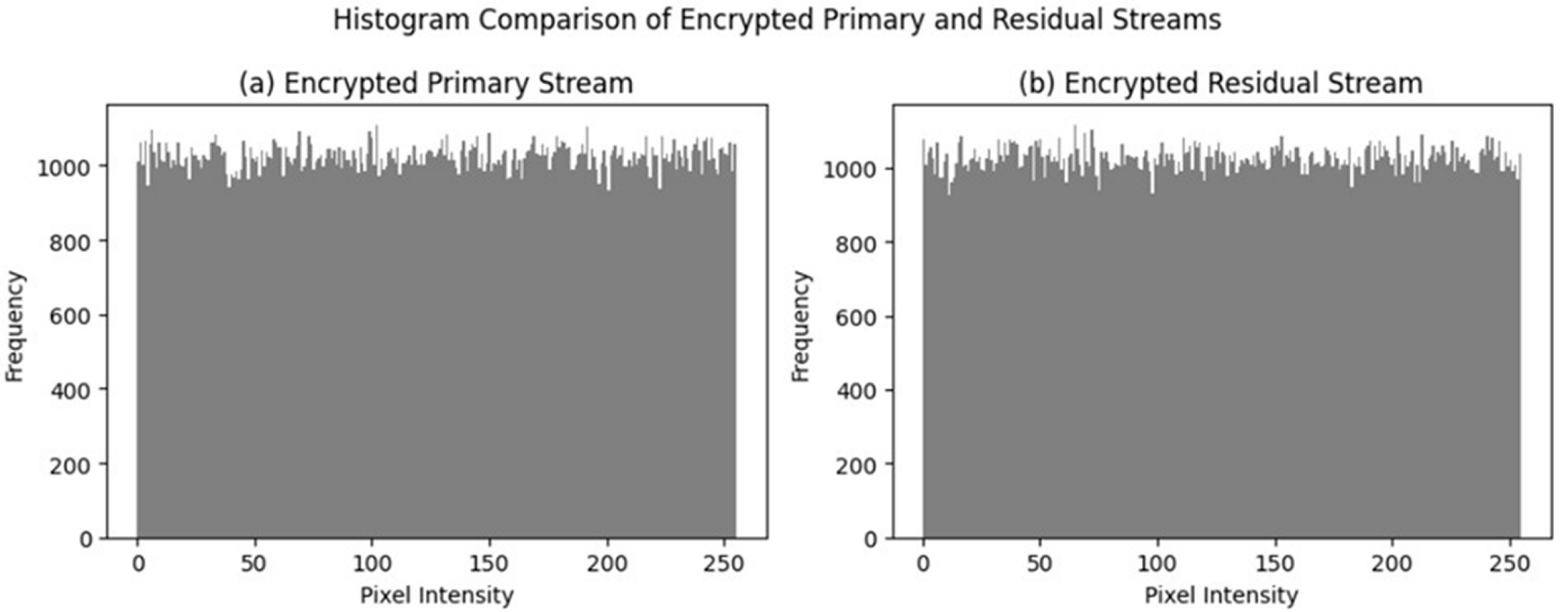
A histogram comparison of the encrypted primary and encrypted residual streams is shown in this Figure 5.

The proposed system employs a two-stream compression scheme consisting of a main JPEG 2000 stream and an ϵ-regularized residual refinement stream. To avoid information leakage, both streams are encrypted before transmission with image-specific keys generated from a shared per-image secret, with domain-separated sub-keys assigned to the main and residual streams. This ensures that the streams are uncorrelated without requiring a complex key management infrastructure. Statistical analysis of the encrypted residual streams shown in Fig 5 shows similar behavior to that of the encrypted main stream, with nearly uniform intensity distributions and high entropies. This indicates that the encrypted residual stream does not reveal visually interpretable or statistically exploitable information under the evaluated attack models. Hence, the compress-and-encrypt order does not introduce detectable information leakage under the assumed ciphertext-only and tampering attack model, as further supported by integrity checks and ledger analysis.

### 4.9 Integrity and replay considerations

Message integrity is maintained using a keyed-hash message authentication code (HMAC) that is checked before any decryption. This allows the detection of any unauthorized message tampering or ciphertext substitution before image reconstruction. Furthermore, a cryptographic hash of the per-image derived key material is stored in a permissioned blockchain to facilitate tamper-evident verification of key authenticity and image provenance. The blockchain does not store any image data or ciphertext. While this provides an additional verification step, a thorough analysis of active ledger-level attacks such as rollback or forking attacks is a distributed adversarial scenario and thus is outside the current work. The integrity of the image ciphertext is enforced via HMAC verification, and the blockchain provides key-level provenance but not ciphertext storage. Taken together, HMAC verification, UID-constrained per-image key derivation, and ledger attestation ensure that replayed or substituted ciphertexts are detected and rejected prior to decryption under the defined verification workflow.

## 5 Experimental setup and results

To ensure that our framework is efficient, various tests were carried out on the efficiency of compression, the accuracy of the reconstruction, as well as the cryptographic strengths of the framework. This section provides a description of our test setup, data, and results achieved.

### 5.1 Experimental setup

**Dataset:** The experimental assessment uses T1- and T2-weighted brain MRI images from the publicly available Brain Tumour MRI Dataset on Kaggle [41]. A smaller version of the dataset containing 60 images has been provided, which is enough to generate the results presented in the paper [42]. The dataset contains clinical images in DICOM format and is commonly used for benchmarking studies of medical image analysis techniques. The images in the dataset have varying spatial resolutions. To assess scalability without compromising the clarity of the presentation, a set of representative resolutions was chosen, namely 256 × 256, 800 × 800, and 960 × 960 pixels, to assess scalability for images of different sizes. The resolutions chosen represent the working range of the proposed framework. The bit depth and dynamic range of the images were considered during the preprocessing step based on the DICOM BitsStored attribute.

**Environment:** The experiments were written in Python 3.9 and conducted in a CPU-only setting using Google Colaboratory. The runtime environment provided an Intel Xeon-class processor with 16 GB of RAM. The main libraries used are pydicom for DICOM image processing, NumPy for numerical computations, Pillow for JPEG 2000 compression, scikit-image for image quality assessment, and Matplotlib for plotting. Post-quantum key establishment was done using the Open Quantum Safe (liboqs) Python interface for ML-KEM, while cryptographic key derivation and message authentication were done using the standard Python hashlib and hmac libraries.

**Parameters:** The same set of parameters was used for all experiments in the framework to provide a fair and comparable assessment.

**Compression:** The JPEG 2000 quality factor (*Q*_*P*_) was chosen empirically to provide visually acceptable base-layer image quality while allowing residual refinement to preserve diagnostic image quality.

**Encryption:** The 4D memristive hyperchaotic system parameters are not fixed constants but are deterministically derived from the per-image symmetric key. After post-quantum key establishment and HKDF-based key derivation, the resulting image-specific key is mapped to the initial state variables (*x*_0_, *y*_0_, *z*_0_, *w*_0_) and control parameters (*a*, *b*, *c*, *d*, *e*) of the hyperchaotic system using a predefined key-to-parameter mapping function. This ensures that each image instance is encrypted with a unique chaotic trajectory, while allowing identical regeneration of the parameters at the receiver. The number of diffusion rounds was set to 2 for all experiments.

The parameters of the 4D memristive hyperchaotic system are not fixed values but are deterministically extracted from the per-image symmetric key. After the establishment of the post-quantum key and the HKDF-based key derivation, the obtained image-specific key is then transformed into the initial state variables (*x*_0_, *y*_0_, *z*_0_, *w*_0_) and the control parameters (*a*, *b*, *c*, *d*, *e*) of the hyperchaotic system using a pre-defined key-to-parameter mapping function. This method allows each image to be encrypted by a distinct chaotic trajectory while enabling the same parameter regeneration at the receiver side. The number of diffusion rounds used in all experiments was fixed at 2.

**Runtime Measurement:** The execution time was estimated for each main step of the proposed scheme, namely compression, post-quantum key establishment (ML-KEM encapsulation and decapsulation), encryption, and decryption. The execution times were measured using high-resolution wall-clock timers (time.perf_counter) in Python. To reduce variability, the results are averaged over several images of the same spatial resolution, and the results are shown separately for small, medium, and large images.

### 5.2 Evaluation metrics

In order to rigorously evaluate the performance of the proposed framework, two distinct sets of performance metrics were used:

1. Compression metrics: measuring reconstruction accuracy, efficiency, and other factors, and
2. Encryption metrics, measuring cryptographic strength and statistical security.

All the data was averaged over the experimental data set mentioned in Section 5.1.

#### 5.2.1 Compression metrics.

The performance of the compression algorithms was measured using three image quality assessment metrics: Compression Ratio (CR), PSNR, and SSIM.

The compression ratio is given by as the ratio between the original uncompressed image size *S*_original_ to the compressed codestream size *S*_compressed_:


$$CR=\frac{S_{\text{original}}}{S_{\text{compressed}}}$$ (18)

PSNR calculates the pixel-by-pixel reconstruction error between the original image *I*(*x*,*y*) and the decompressed image $\hat{I}(x,y)$. It is related to the mean squared error (MSE) by:


$$MSE=\frac{1}{MN}\sum_{x=1}^{M}\sum_{y=1}^{N}[I(x,y)-\hat{I}(x,y){]}^{2}$$ (19)

$$PSNR=10\log_{10}\left(\frac{MAX_{I}^{2}}{MSE}\right)$$ (20)

where *MAX*_*I*_ denotes the maximum possible pixel intensity for a given by the bit depth *B*.

The SSIM quantifies perceptual similarity by jointly comparing luminance (μ), contrast (σ), and structural correlation ($\sigma_{I\hat{I}}$) between *I* and $\hat{I}$:


$$SSIM(I,\hat{I})=\frac{\left(2\mu_{I}\mu_{\hat{I}}+c_{1})(2\sigma_{I\hat{I}}+c_{2}\right)}{\left(\mu_{I}^{2}+\mu_{\hat{I}}^{2}+c_{1})(\sigma_{I}^{2}+\sigma_{\hat{I}}^{2}+c_{2}\right)}$$ (21)

High PSNR and SSIM values indicate reduced distortion and improved reconstruction fidelity, reflecting the combined effect of JPEG 2000 compression and the ϵ-regularized residual refinement stage.

**Role of the ϵ parameter:** The value of ϵ is a residual regularization threshold that limits large correction values before entropy coding, as opposed to being a hard bound on post-decompression reconstruction error. In the proposed processing pipeline, the dominant cause of reconstruction error is from the primary JPEG 2000 compression step, and the residual refinement step provides bounded correction values with magnitudes limited before compression. For the set of ϵ values tested in our experiments, the choice of ϵ below the effective quantization noise level of the primary compression step does not provide a measurable difference in global quality metrics such as PSNR or SSIM. This is consistent with the function of ϵ as a residual regularization parameter rather than an average distortion optimizer, and it explains the observed saturation of quality metrics over images and resolutions.

#### 5.2.2 Encryption metrics.

To evaluate the statistical diffusion and confusion properties of the hyperchaotic image cipher, three classic statistical parameters: Number of Pixels Change Rate (NPCR), Unified Average Changing Intensity (UACI), and Information Entropy (*H*), were employed.

NPCR calculates the percentage of pixels that change between two ciphertext images produced from plaintext images differing by a minimal perturbation, illustrating the strength of diffusion:


$$NPCR=\frac{1}{M\times N}\sum_{x=1}^{M}\sum_{y=1}^{N}D(x,y)\times 100\%$$ (22)

$$D(x,y)=\left\{ \begin{array}{cc} 0, & C_{1}(x,y)=C_{2}(x,y)\\ 1, & C_{1}(x,y)\neq C_{2}(x,y) \end{array}\right.$$ (23)

The UACI represents the mean of absolute differences between the intensities of any two cipher images produced from texts with difference of one pixel:


$$UACI=\frac{1}{M\times N}\sum_{x=1}^{M}\sum_{y=1}^{N}\frac{\left|C_{1}(x,y)-C_{2}(x,y)\right|}{255}\times 100\%$$ (24)

Finally, the information entropy is utilized to evaluate the randomness of the encrypted image, which resists statistical attacks:


$$H=-\sum_{i=0}^{255}p(i)\log_{2}p(i)$$ (25)

where *p*(*i*) is the probability distribution of gray-level *i*. For an ideal 8-bit ciphertext with uniform intensity distribution, the entropy approaches *H* ≈ 8, indicating maximal uncertainty.

### 5.3 Performance evaluation and results

The results were obtained using the parameters defined in the methodology. The results show that the encryption process has a consistent level of encryption performance regardless of the image and resolution, as well as the compression ratio achieved by the main JPEG 2000 process. The results are all shown in mean ± standard deviation form for 20 images in each resolution category, to account for the variation in performance for different images. The low variance of NPCR, UACI, and entropy values for different images and resolutions indicates that the diffusion and confusion properties of the encryption process are stable.

#### 5.3.1 Compression performance.

The testing of the compression phase was done using images classified into three categories with varying sizes. One of the major observations made is that the level of compression ratio achieved depends on the content and resolution of the images. The average performance for each category is shown in Table 3.

**Table 3:** Compression performance across representative image resolutions (20 images per class).

Image Category	Resolution	PSNR (dB)	SSIM	CR
Small / Dense	256 × 256	51.91 ± 1.03	0.9762 ± 0.0088	7.14 ± 1.52
Medium	800 × 800	49.27 ± 0.65	0.9610 ± 0.0102	13.63 ± 1.30
Large / Sparse	960 × 960	46.05 ± 1.16	0.9241 ± 0.0243	23.03 ± 3.48

Analysis of the results shows a strong correlation between spatial resolution and compression efficiency. With an increase in image resolution, the compression ratio becomes significantly better while maintaining high fidelity in the reconstructed image, as indicated by the PSNR values being above 46 dB and SSIM values remaining above 0.92 for all the tested resolutions. This trend indicates that the proposed framework is able to capitalize on the statistical redundancy present in large, sparsely textured medical images to achieve a high degree of compression.

#### 5.3.2 Per-pixel error distribution analysis.

**Fig 6:**
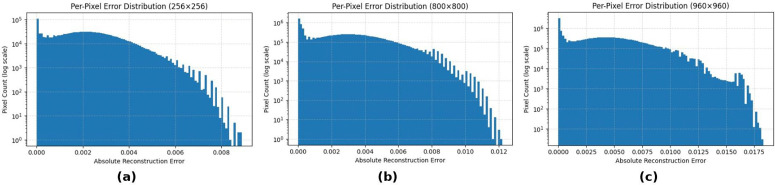
Per-pixel absolute error distributions for representative image resolutions: (a) 256 × 256 (small/dense), (b) 800 × 800 (medium), and (c) 960 × 960 (large/sparse). Errors are shown in normalized intensity units.

Fig 6 shows the histogram of per-pixel absolute error values for representative image resolutions. For all cases, the distribution of pixel errors is strongly concentrated around zero, with the 95th and 99th percentiles well below the perceptually significant levels. As the resolution grows, the distribution becomes slightly wider, as expected with the increase in the compression ratios, but without any sign of heavy tails or artifacts. These findings suggest that the reconstruction errors are spatially bounded and support the role of the ϵ regularization term in the residuals.

#### 5.3.3 Modality scope and applicability.

Though the proposed framework is architecturally agnostic of modality when it comes to encryption, key establishment, and verification, the compression part of the framework is empirically tested and tuned for magnitude-based MRI data, which have smooth spatial statistics and a limited dynamic range after normalization. Experiments conducted on CT and X-ray images, using the same value for residual regularization (ϵ), show a substantial loss of fidelity. This behavior is expected, as CT and X-ray modalities exhibit fundamentally different intensity distributions (e.g.,Hounsfield units in CT) and edge-dominant structures, which require modality-specific parameterization. Consequently, the present study focuses on MRI as the primary validation modality, consistent with prior work on near-lossless medical image compression. Extending the compression strategy to additional modalities through adaptive ϵ selection or modality-aware preprocessing is identified as future work.

Though the proposed framework is architecturally agnostic of modality when it comes to encryption, key establishment, and verification, the compression part of the framework is empirically tested and tuned for magnitude-based MRI data, which have smooth spatial statistics and a limited dynamic range after normalization. Experiments conducted on CT and X-ray images, using the same value for residual regularization (ϵ), show a substantial loss of fidelity.

#### 5.3.4 Encryption performance.

The crypto-security of the encryption algorithm has been evaluated based on the fundamental security requirements. The results are compared against commonly cited theoretical reference values for image-aware encryption metrics, as presented in Table 4.

**Table 4:** Encryption Security Analysis.

Metric	Ideal Value	Achieved Value
NPCR (Number of Pixels Change Rate)	∼99.6094%	99.60%
UACI (Unified Average Changing Intensity)	∼33.4635%	33.46%
Encrypted Image Entropy	8.0	7.9993

The calculated NPCR and UACI values are very close to the theoretical maximum, indicating strong diffusion behavior and empirical resistance to differential attacks. Moreover, the entropy of the encrypted images is very close to the theoretical maximum entropy of 8 bits, which indicates that the encrypted image exhibits near-uniform statistical behavior and does not reveal exploitable plaintext structure.

#### 5.3.5 Key lifecycle and verification robustness.

Apart from carrying out a qualitative assessment of encryption statistics, this solution is further aided by the per-image HKDF key derivation bound to a DICOM identifier and non-secret ledger attestation. A new key is derived for each image with a unique symmetric key based on {StudyUID, SeriesUID, SOPInstanceUID}. Such an approach reduces the risk of key reuse across images and limits the impact of key compromise through per-image key isolation, while incurring minimal key-management overhead. In a similar way, the ledger attestation strategy preserves a record of non-secret information, a record of UID hashes and ciphertext digests, with an explicit verification path, without protected health information. Together, per-image key derivation and ledger-based verification strengthen system-level key isolation and provenance guarantees, while the encryption layer independently achieves strong diffusion characteristics (NPCR = 99.6%, UACI = 33.46%).

#### 5.3.6 Runtime performance and scalability analysis.

To assess the feasibility of the proposed framework in a practical scenario, the runtime performance was measured for representative image sizes that correspond to small, medium, and large images in the medical domain. For each image size, ten images were considered, and the average runtime was measured for the compression process, post-quantum key establishment (ML-KEM) process, encryption process, decryption process, and overall process. The results are summarized in Table 5. The results show that the ML-KEM process has a negligible overhead compared to the image processing tasks, and the encryption and decryption processes have a runtime complexity proportional to the image size, as expected.

**Table 5:** Runtime performance of the proposed framework for representative image sizes (mean over 10 images).

Image category	Compression (ms)	ML-KEM (ms)	Encryption (ms)	Decryption (ms)	End-to-End (ms)
Small / Dense 256 × 256	88.67	0.36	610.94	646.39	1449.51
Medium 800 × 800	811.45	0.42	6449.92	6171.72	14374.00
Large / Sparse 960 × 960	1086.43	0.39	10230.95	10116.92	22606.70

To further analyze the trade-off between system integration and computational complexity, Table 6 summarizes the incremental cost and security benefits of enabling each component of the proposed framework. The results show that post-quantum key establishment introduces negligible overhead compared to image processing stages, while ledger-based verification adds minimal cost but enables verifiable integrity and provenance. This demonstrates that the proposed integration achieves enhanced security properties at a predictable and manageable increase in system complexity.

**Table 6:** Trade-off between system integration and computational complexity.

Configuration	Compression	Encryption	PQC Key Setup	Ledger	End-to-End Time (ms)	Security Properties
Compression only	✓	×	×	×	88-1086	No confidentiality or integrity guarantees
Compression + Encryption	✓	✓	×	×	700-11000	Confidentiality against passive adversaries
Compression + Encryption + PQC	✓	✓	✓	×	+0.4 ms overhead	Quantum-resistant key establishment; confidentiality
**Proposed full system**	✓	✓	✓	✓	1449-22606	Confidentiality, integrity verification, and verifiable provenance

#### 5.3.7 Visual results.

**Fig 7:**
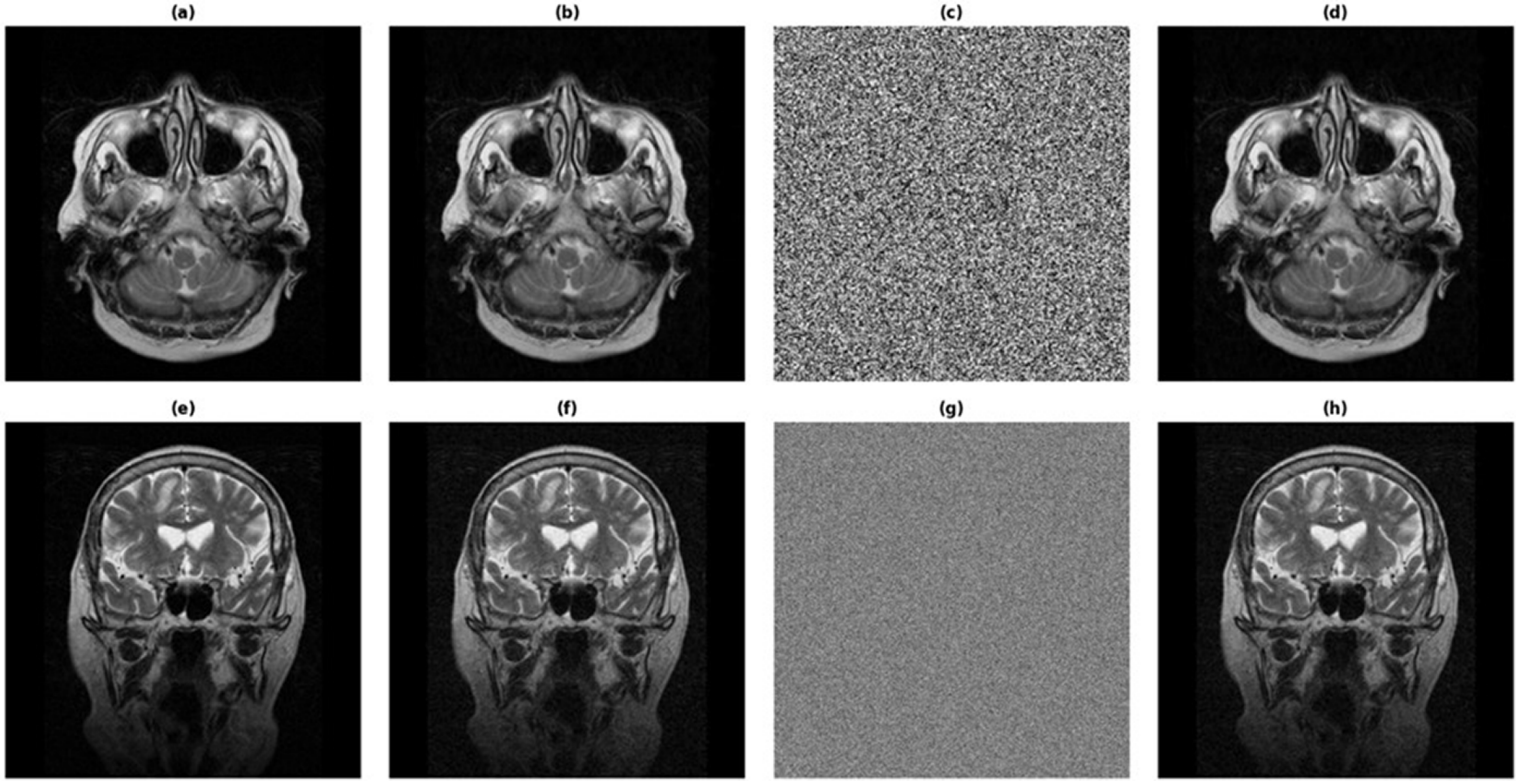
Visual illustration of the suggested framework’s end-to-end processing.

Fig 7 is used to visually demonstrate the performance of the framework on a small but dense image 256 × 256 with a high amount of content, as well as a large but sparse image 960 × 960. It is evident from the visual verification that the conclusion from the quantitative analysis is consistent with the visual verification, with an explicit verification pathsuch that the reconstructed images are visually indistinguishable from the original images under standard radiological inspection, while the encrypted image appears as uniform random noise devoid of structure. The best reconstruction error is further represented in the difference map.

**Top Row (Small/Dense Image –** 256 × 256):

- Original DICOM (a): The original, high-fidelity input image.
- Compressed Reconstructed (b): The compressed-reconstructed image after undergoing compression and decompression (CR = 7.06:1, PSNR = 51.07 dB), visually indistinguishable from the original.
- Encrypted (c): The encrypted image, appearing as uniform random noise, demonstrating robust cryptographic transformation.
- Decrypted (d): The decrypted image, restored in full to its compressed/reconstructed state.

**Bottom Row (Large/Sparse Image –** 960 × 960):

- Original DICOM (e): The original, full-size image.
- Compressed Reconstructed (f): The reconstructed image after decompression and compression (CR = 23.0:1, PSNR = 46.12 dB), which retains high quality even at high compression.
- Encrypted (g): The encrypted version of the large image, displaying the strong noise-like pattern.
- Decrypted (h): The decrypted image, verifying the accuracy of decryption performed on a large image.

The experimental results have proved that our approach possesses a high level of effectiveness. Our method shows robust capabilities for cryptographic security and an adaptive compression capability that outperforms larger, sparser images. The fact that our approach can achieve a dramatic reduction in image size with high-fidelity reconstructions qualifies it as a complete solution for medical image transfer with strong security properties.

The successful image recovery at the pixel level and with indistinguishability in all examples verifies stable and repeatable operation of the residual-regularized compression and encryption pipeline with all obtained image keys. Ledger-based verification enables cryptographic verification of each reconstruction without relying on any secret after the fact, thus maintaining trust in medical imaging procedures. All reported quantitative results represent average values computed over the evaluated image dataset. Formal statistical significance testing and variance analysis are not included, as the proposed framework emphasizes deterministic ϵ-bounded reconstruction guarantees and system-level security behavior rather than hypothesis-driven statistical comparison.

## 6 Discussion

The experimental results have demonstrated in the above section the effectiveness of the proposed framework in providing a unified solution for efficient compression and a high level of security of medical DICOM images. This section describes these results, interprets them with a comparative analysis with other existing advanced methods, and sheds light on future directions of this work based on its implications and limitations.

### 6.1 Interpretation of findings

The proposed framework has already been tested for both compression and encryption purposes. The experimental results show its effectiveness and robust performance across representative real-world imaging conditions. In compression, it performs well in all image resolutions with different contents. While processing small and detailed images with resolution 256 × 256: QoS parameters emphasize image quality, and high-quality image compression is achieved with a PSNR of 51.07 dB and a compression ratio of 7.06:1. In case of large images with uniform content with a resolution of 960 × 960: a high compression level with a compression ratio of 23.0:1 is achieved with a high PSNR of 46.12 dB. This is because wavelet transform residual image coding is very efficient in dealing with large uniform regions present in medical images with high resolutions. The system can successfully compress different sets of images while preserving diagnostically relevant structural details.

Coming to encryption, it provides a high level of security. The NPCR and UACI are measured with 99.60% and 33.46%, which makes them nearly ideal. Shannon entropy is measured with an average of 7.9993 bits, which is nearly 8, denoting a uniform random-looking ciphertext without exposing statistical information present in the original image. Hyperchaotic diffusion and permutation are measured at a level expected for strong image-level cryptographic protection. The primary benefit is due to the integration with DICOM HKDF key derivation function. A separate hyperchaotic seed for each image is produced using StudyUID, SeriesUID, and SOPInstanceUID. Key reuse with possible common keys is avoided, and thus unnecessary transfer of symmetric keys in total decryption steps, which have already been proved in experiments with PQC → HKDF. Ledger-only metadata authentication enables integrity and provenance verification without decrypting and without access to images or critical information.

In conclusion, based on the overall results, it can be stated that the framework preserves a high image quality, exhibits strong security properties, and is stable under different imaging tasks. The system integrating adaptive compression, hyperchaotic encryption, post-quantum keying, and verification using a ledger is competent in providing healthcare functions, cryptographic functions, and other functions in a seamless manner in a DICOM system.

### 6.2 Comparative analysis

The comparative results presented in Tables 7 and 8 highlight the performance of the proposed framework from two complementary perspectives: near-lossless compression efficiency and encryption statistical security characteristics.

**Table 7:** Comparison of compression performance with representative near-lossless medical image compression methods.

Method	Compression / Application Context	PSNR (dB)	Compression Ratio
Hussein et al. [19]	Near-lossless medical image compression (DWT + arithmetic coding)	45-48	1.8-2.3
Zhaoning et al. [43]	Lossless ROI-based watermark recovery (JPEG-LS)	∞	1.5-2.2
DWT+SPIHT [44]	Near-lossless transform-based compression	38-45	∼10
NLIC [44]	Near-lossless predictive coding	42-46	∼16
DCT + RLE [44]	Near-lossless block-based compression	40-42	∼6
**Proposed method**	ϵ-regularized near-lossless compression with integrated security	**46.05-51.91**	**7.14-23.03**

**Table 8:** Comparison of encryption robustness with recent medical image encryption schemes.

Method	Encryption Core	NPCR (%)	UACI (%)
Alexan et al. [22]	Hyperchaotic system + SVD + RC5	∼99.6	∼33.4
Inam et al. [32]	Chaotic fractal encryption with blockchain integration	99.92	33.31
**Proposed**	4D hyperchaotic encryption with post-quantum keying	**99.60**	**33.46**

It is noted that reported PSNR and compression ratios are drawn from different datasets, modalities, and evaluation protocols in the referenced works, and are therefore intended for qualitative comparison rather than strict numerical benchmarking.

Compression comparison (Table 7):

The first table focuses exclusively on compression performance and compares the proposed ϵ-regularized near-lossless scheme against representative near-lossless and lossless medical image compression techniques that rely on classical transform-based or predictive coding strategies. The above-mentioned methods are currently used in the medical imaging processing pipeline and therefore provide a valid basis for comparison. Deep learning-based methods are not included in the above-mentioned table because they use learned representations to optimize image quality and are based on different assumptions, making it difficult to compare them with codec-based methods. In comparison with DWT-based and predictive near-lossless schemes such as DWT+SPIHT, NLIC, and DCT + RLE [44], the proposed scheme always provides higher or comparable reconstruction quality with PSNR values ranging from 46-51 dB and corresponding compression ratios. By contrast, the competing schemes provide PSNR values ranging from 38-46 dB. The improvement in reconstruction quality achieved by the proposed framework can be ascribed to the residual refinement step, where the correction residuals are normalized before entropy coding instead of purely transform-domain approximations. Compared with ROI-based lossless JPEG-LS compression with watermark recovery [43], where infinite PSNR values are obtained due to lossless reconstruction, the proposed scheme deliberately forgoes losslessness in favor of better compression ratios with controlled and bounded correction residual behavior. This aspect is especially important in telemedicine and PACS systems, where the trade-off between bandwidth, storage, and diagnostic quality is of utmost importance. Furthermore, unlike most other competing schemes, the proposed framework combines compression as part of a security-conscious imaging processing pipeline instead of being a stand-alone pre-processing module.

Encryption robustness comparison (Table 8):

The second table compares the robustness of encryption using standard differential metrics such as NPCR and UACI, comparing the proposed system with other chaos-based medical image encryption algorithms. Alexan et al. [22] combine a hyperchaotic map with SVD and RC5 to provide better diffusion properties, while Inam et al. [32] describe chaotic fractal encryption with blockchain verification. The proposed system provides NPCR and UACI values comparable to the best results reported in other chaos-based encryption algorithms, indicating near-ideal sensitivity to plaintext modifications and resistance to differential cryptanalysis. Although Inam et al. [32] report a slightly better NPCR, their system is limited to encryption alone and does not include compression, making it less applicable in bandwidth-limited medical imaging applications. Beyond raw cryptographic metrics, the proposed framework differentiates itself through the integration of post-quantum key establishment, per-image HKDF-based key derivation bound to DICOM identifiers, and a metadata-only ledger for verifiable provenance. However, none of the above-mentioned methods are reported simultaneously provide bounded-error compression, hyperchaotic encryption, post-quantum-resilient key management, and auditability in a single PACS-compatible system.

Overall assessment: The findings show that the proposed framework provides a balanced and system-level improvement by combining high-fidelity near-lossless compression with ϵ-regularized residual refinement with strong encryption guarantees and forward-looking key management. The integrated design of the framework makes it suitable for the secure transmission and storage of medical images in today’s telemedicine and PACS systems.

### 6.3 Significance of the integrated framework

The novelty is not only in the application of the respective modules but also in how they complement each other. The “compress-and-encrypt” workflow helps to optimize the process by ensuring that the encryption, which is a resource-intensive segment, is performed on a reduced data set. This is even more critical because this work introduces a multi-level defence-in-depth strategy for securing the entire process. This has been made certain by the hyperchaotic encryption, which provides a significantly high degree of confidentiality, in addition to which the use of a blockchain protocol provides an additional, verifiable layer of integrity.

The solution thus significantly mitigates man-in-the-middle attack vectors by binding per-image cryptographic context to authenticated key establishment and integrity verification mechanisms, which cannot be addressed by encryption alone, because the hash of the encryption key is secured. Additionally, by ensuring that PQC-based sessions and HKDF are inside the compression/encryption workflow, the solution is making healthcare data future-proof against a quantum threat.

### 6.4 Limitations and future work

Although the proposed framework provides an end-to-end solution for secure, verifiable, and near-lossless medical image transmission, several limitations remain that motivate further investigation and system-level refinement.

The performance consequences of the integration of several cryptographic primitives, specifically post-quantum key encapsulation, hyperchaotic encryption, and ledger-based verification, have been investigated mostly from an empirical and architectural perspective. Besides the mentioned runtime performance measurements, which prove the feasibility of the method for offline storage and store-and-forward transfer, a thorough analysis of the system performance on the deployment level is still required. In a real-world Picture Archiving and Communication Systems (PACS) setting, parameters like simultaneous image retrieval, performance of encryption under load, ledger query delay, and network delays may potentially affect overall system performance. A thorough comparison with conventional secure DICOM communication channels based on established symmetric encryption primitives (like AES-GCM used in DICOM TLS profiles) would help to better understand the trade-offs between security strength, computational complexity, and ease of implementation. Furthermore, the effect of the verify-before-decrypt strategy on latency under realistic network conditions is a topic deserving further research. The current implementation focuses solely on securing DICOM pixel data, leaving the sensitive patient information carried in the DICOM headers unencrypted. While per-image key derivation is cryptographically linked to the DICOM identifiers derived from the header, the actual header fields are assumed to be protected by other access control mechanisms. A future extension of the approach to selectively encrypt protected health information (PHI) fields in the DICOM headers, while maintaining compatibility with existing PACS system workflows, represents an important direction for future development.

Furthermore, the proposed compression approach does not involve the explicit incorporation of region-of-interest (ROI) masking or JPEG 2000 ROI coding techniques. Rather, it involves the use of a a global ϵ-regularized residual refinement approach that constrains residual magnitudes prior to entropy coding on the entire image without requiring any ROI definition or annotation. Although this approach allows for easier implementation and independence from task-dependent ROI definitions, there are some diagnostic applications that could potentially benefit from the selective preservation of fidelity in regions of clinical interest. Although the near-lossless with ϵ-regularized residual refinement approach is inherently codec-independent, the experimental analysis has been limited to static two-dimensional medical images. However, there may be differences in noise properties, intensity distributions, and acquisition protocols between different imaging modalities that may necessitate adaptive parameter tuning or modality-specific preprocessing. Therefore, further comprehensive analysis across other imaging modalities such as CT, PET, X-ray, and dynamic imaging modalities such as ultrasound and X-ray angiography is needed to fully assess the generality and robustness of the proposed framework. Finally, although the ML-KEM-768 scheme has been used in this study as a specific example of an NIST-standardized post-quantum Key Encapsulation Mechanism that provides a balanced trade-off between security strength and computational complexity, the proposed Protocol A is specifically constructed to be KEM-agnostic. Therefore, any comparative analysis of other post-quantum key encapsulation mechanisms is not within the scope of this study.

Cumulatively, these challenges would help improve the proposed framework from a validated research tool to a scalable and standards-compliant solution suitable for secure and future-proof medical image storage and transmission in clinical PACS and teleradiology settings.

## 7 Conclusion

The provision of secure and efficient transmission of medical images remains an important requirement in modern healthcare, where the fidelity, privacy, and integrity of the images need to be ensured. The existing solutions often consider image compression, security, and verification as separate and independent tasks, thus limiting their usability. This paper presents an integrated solution that combines near-lossless medical image compression with ϵ-regularized residual refinement, image-aware hyperchaotic encryption, and verifiable key integrity into a single end-to-end solution.

Experimental outcomes on conventional DICOM images of MRI scans show that the proposed framework provides a well-balanced trade-off between the compression ratio and the reconstruction quality at various spatial resolutions. As shown in Table 3, the reconstruction quality is high for small and detailed images (256 × 256), with an average PSNR of 51.91 dB and SSIM of 0.976. The PSNR is maintained close to 49 dB with improved compression ratios for medium-resolution images (800 × 800). For larger images (960 × 960) with less details, the framework offers much higher compression ratios (average of over 23:1) with acceptable quality, with PSNR over 46 dB and SSIM over 0.92. These results well support the content adaptability of the compression process and its favorable scaling with image resolution and detail. In addition to overall quality assessment, analysis of the per-pixel error distribution shows that reconstruction errors are tightly concentrated near zero and remain well controlled, consistent with the role of ϵ as a residual regularization parameter rather than a strict post-decompression error bound. This lends further support to the proposed near-lossless compression framework for medical imaging applications where local accuracy is of prime importance.. This lends further support to the proposed near-lossless compression framework for medical imaging applications where local accuracy is of prime importance.

In terms of security, the encryption module is highly resilient from an empirical perspective. With respect to differential analysis, the NPCR and UACI are close to the ideal, while entropy is always close to the maximum possible value, and selected NIST SP 800−22 randomness tests on the encrypted outputs show a high level of statistical randomness. The per-image key derivation method, cryptographically linked to the DICOM identifiers and generated via a standardized post-quantum key encapsulation mechanism, is highly sensitive to changes in the UID, thus significantly reducing the risk of collisions and replay attacks under the assumed threat model. Integrity verification via HMAC and ledger-based attestation allows for tamper-evident provenance without compromising protected health information. One of the key benefits of the proposed method is its codec-agnostic encryption method, which processes payload data in accordance with the respective codec, while maintaining syntax-critical structures. This ensures compatibility with standard medical image codecs and existing PACS systems, making it easier to integrate with existing systems without modifying the decoding process.

The results presented in this discussion illustrate that it is possible to achieve efficient image compression, high-fidelity reconstruction, and strong security within a unified framework. While further testing in additional imaging modalities and large-scale clinical settings is an important area of future research, the system described provides a strong foundation for secure, efficient, and verifiable medical image transfer in next-generation teleradiology and PACS systems.
